# Removal efficacy of fly ash composite filler on tailwater nitrogen and phosphorus and its application in constructed wetlands

**DOI:** 10.3389/fchem.2023.1160489

**Published:** 2023-04-19

**Authors:** Shuhang Wang, Haoran Yang, Feifei Che, Wei Huang, Dianhai Yang

**Affiliations:** ^1^ State Key Laboratory of Pollution Control and Resource Reuse, College of Environmental Science and Engineering, Tongji University, Shanghai, China; ^2^ State Environmental Protection Engineering Center for Pollution Treatment and Control in Textile Industry, College of Environmental Science and Engineering, Donghua University, Shanghai, China; ^3^ National Engineering Laboratory for Lake Pollution Control and Ecological Restoration, Institute of Lake Environment and Ecology, Chinese Research Academy of Environmental Sciences, Beijing, China

**Keywords:** tailwater, filler, nitrogen removal, phosphorus removal, constructed wetlands

## Abstract

Constructed wetlands (CWs) have been widely used in tailwater treatment. However, it is difficult to achieve considerable removal efficiency of nitrogen and phosphorus in tailwater solely by CWs—an efficient green wetland filler is also important. This study investigated 160 domestic sewage treatment facilities (DSTFs) in rural areas from two urban areas in Jiaxing for TP and NH_3_-N and found that TP and NH_3_-N concentrations in rural domestic sewage (RDS) in this plain river network are still high. Therefore, we selected a new synthetic filler (FA-SFe) to enhance nitrogen and phosphorus reduction, and we discuss the importance of filler in constructed wetlands. Experiments revealed the adsorption capacity of the new filler: the maximum adsorption amounts of TP and NH_3_-N reached 0.47 g m^-2^ d^-1^ and 0.91 g m^-2^ d^-1^, respectively. The application potential of FA-SFe was verified in actual wastewater treatment, with the removal rates of ammonia nitrogen and TP reaching 71.3% and 62.7%, respectively. This study provides a promising pathway for nitrogen and phosphorus removal from rural tailwaters.

## 1 Introduction

Rural domestic sewage (RDS) has played a significant role in surface water environments in recent years (Wu et al., 2011; Wang et al., 2019). Rural area water environments are not only related to living conditions or health security but are also a vital part of water quality in urban water environments. Total phosphorus (TP), ammonia nitrogen (NH_3_-N), and chemical oxygen demand (COD) are the main chemical indices of rural domestic sewage (RDS), and pollutant discharge has direct and indirect effects on the quality of surface water environments (Mukherjee et al., 2022). RDS is an important part of rural water environment systems, and its treatment has become an important way of improving surface water environments (Cheng et al., 2020; Li et al., 2020).

The combination of anaerobic–anoxic–oxic (A^2^/O) and constructed wetlands (CWs) has been widely used to treat RDS. The maximum removal efficiency of COD could be as high as 80%–90% (He et al., 2017; Ma et al., 2019; Liu et al., 2020; Zhao et al., 2020), achieving local wastewater emission standards. However, NH_3_-N and TP removal efficiency has been relatively low due to a lack of high process performance, systematic facility operation, or regulation (Li et al., 2015; Zhang et al., 2017; Li et al., 2018). CWs play a significant role in NH_3_-N and TP removal, especially for TP. Plants and fillers have been the main components of CWs. If the same plants were used in CWs, then fillers could become the key factor in efficient TP removal (Zhao et al., 2019; Mateus and Pinho, 2020). Appropriate fillers not only have high removal efficiency for COD but can also increase the removal efficiency of TP and NH_3_-N to improve the performance of CWs (Banerjee et al., 2022).

Surface flow-constructed wetlands (SF-CWs) have low-cost investment advantages, convenient management, and large storage capacity. Water environment health and ecological restoration in river or lake basins could also be influenced (Maucieri et al., 2017). However, it has been difficult to reach ideal efficiency in pollutant removal. TP and NH_3_-N removal efficiency in practical engineering applications has been less than 50% and 60%, respectively, due to magnification effects, geography, climate, seasons, and filler saturation (Reddy et al., 2001; Liu et al., 2016; Wang et al., 2018). It has been found that CWs that used *Typha latifolia* for RDS treatment had low TP and TN efficiencies of 11% and 18%, respectively (Saha et al., 2015). Coarse gravel aggregates were also used with *Cyperus papyrus* in CWs to remove nutrients, with a TP removal efficiency of only 4% (Bateganya et al., 2016). In addition, different types of CWs have various target pollutants (Chowdhury et al., 2019). Generally, with a combination of vertical subsurface flow (VF) and horizontal subsurface flow (HF), CWs exhibit more efficiency in removing nitrogen and phosphorus (Vymazal, 2007; Wu et al., 2011). However, reaching theoretical pollutant removal efficiency is challenging, and the application costs are high.

In the Jiaxing plain river network, urbanization processes are rapid with frequent human activity, and most RDS treatments use different processes due to the area’s developed economy (Ruhl et al., 2022). However, the low removal efficiency from RDS of several pollutants (such as TP and NH_3_-N) using domestic sewage treatment facilities (DSTF) is this area's main problem. It is difficult for DSTF effluent treatment to achieve local wastewater emission standards, with the nutrient in effluent entering the regional water environment system, which can influence the surface water environment (Qu et al., 2018; Shukla et al., 2020). In addition, the high proportion of soluble reactive phosphorus (SRP) in RDS causes low TP removal efficiency when traditional treatment processes (A/O or A^2^/O) are used (Khorshid et al., 2019; Qin et al., 2019). Therefore, CWs are widely used in RDS treatment, so choosing fillers is very significant.

Increasing the performance of CW fillers is the main way of improving RDS treatment (Mukherjee et al., 2023). This study investigated the spatial and temporal distribution of pollutants from 160 DSTFs in the Jiaxing plain river network and chose fly ash as the new filler; this was utilized in ten DSTFs. The TP and NH_3_-N removal efficiency and mechanisms were studied using the new fillers in CWs, and subsequent CW performance was evaluated. The results will provide technical support for RDS treatment.

## 2 Methods and materials

### 2.1 Sampling sites and collection

**FIGURE 1:**
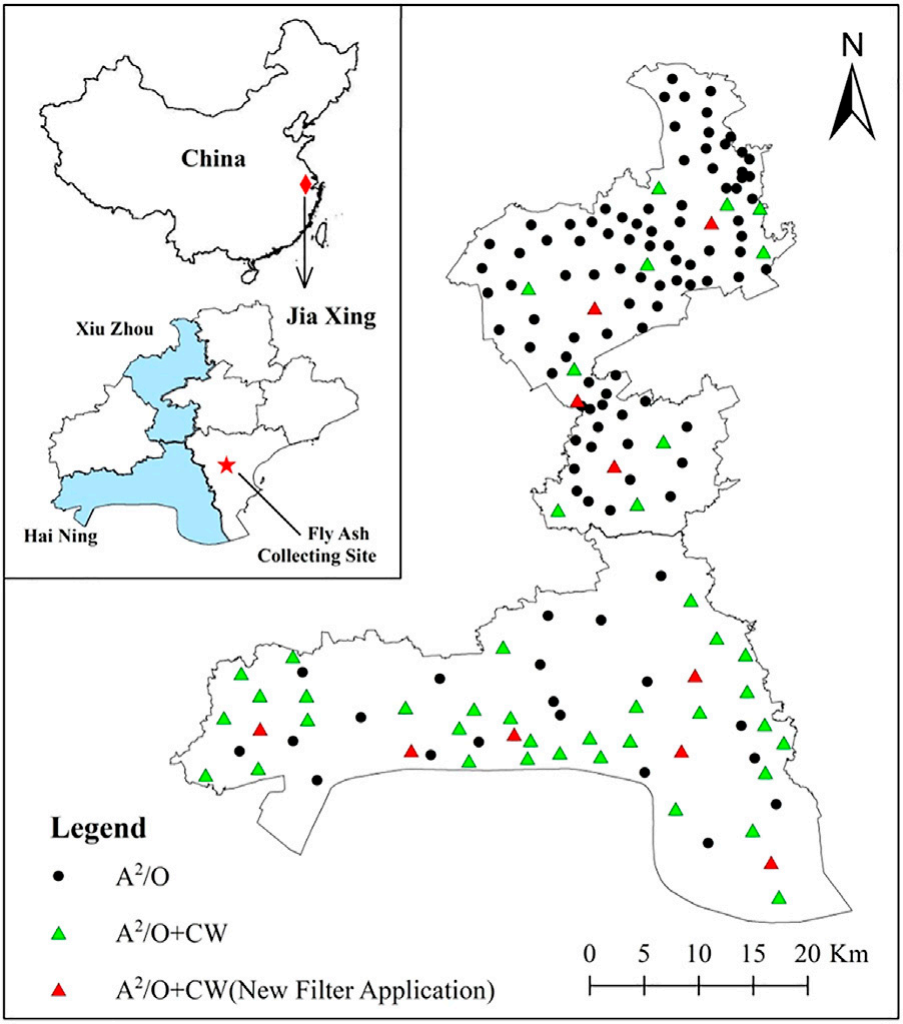
Sites of 160 DSTFs in the rural area.

A total of 160 DSTFs (63 A^2^/O + CW and 97 A^2^/O processes) in the rural area were chosen for this study from two country-level cities in the Jiaxing plain river network. The scales of the 160 DSTFs are ≥30 m^3^ d^-1^, and the sampling sites are shown in Figure 1. The influent and effluent of the 160 DSTFs over four seasons were collected in September 2018 (autumn), December 2018 (winter), March 2019 (spring), and June 2019 (summer). The water samples were taken to the laboratory within 24 h for physicochemical parameter measurement.

### 2.2 Sorption and kinetics experiments

Fly ash (FA), collected from a local factory, was chosen as the new filler for the CWs (Figure 1). Then 10.0 g FA was added to 1 L of 3.0 mol L^−1^ H_2_SO_4_ solution, and the resulting mixture was shaken for 12 h at room temperature (25°C ± 2°C). Modified FA was obtained by filtration and drying and was labeled “FA-S.” The other two modified FAs were obtained by adding 10.0 g FA and FA-S to 1 L of 0.2 mol L^−1^ FeSO_4_ solution, respectively, and the mixtures were shaken for 12 h at room temperature (25°C ± 2°C). The Fe-doped fillers (FA-Fe and FA-SFe) were obtained by filtration and drying.

SRP and NH_3_-N sorption kinetics were examined in a solution with initial SRP and NH_3_-N concentrations of 2 mg L^-1^ and 10 mg L^-1^, respectively. The raw FA and new fillers (2 g) were added to a series of conical flasks (220 rpm, room temperature) and mixed with 100 mL SRP and NH_3_-N solution. At 11 different time intervals (1, 2, 5, 10, 20, 40, 60, 120, 240, 360, and 480 min), suspensions were obtained from each flask and then centrifuged, filtered (0.45 um), and analyzed for SRP and NH_3_-N concentration.

The sorption isotherm of the fillers was obtained by using batch experiments. Fillers (FA, FA-S, FA-Fe, and FA-SFe) of 2 g were added to 100 mL SRP and NH_3_-N solution at different concentrations (0, 0.1, 0.5, 1, 2, 5, and 10 mg L^-1^ as KH_2_PO_4_ and 0, 0.1, 0.5, 1, 2, 5, and 10 mg L^-1^ as NH_4_Cl, respectively). They were continuously agitated in a shaker at 220 rpm at room temperature for 480 min. The suspensions were centrifuged, filtered (0.45 um), and analyzed to determine SRP and NH_3_-N concentration.

### 2.3 New filler application in CWs

**FIGURE 2:**
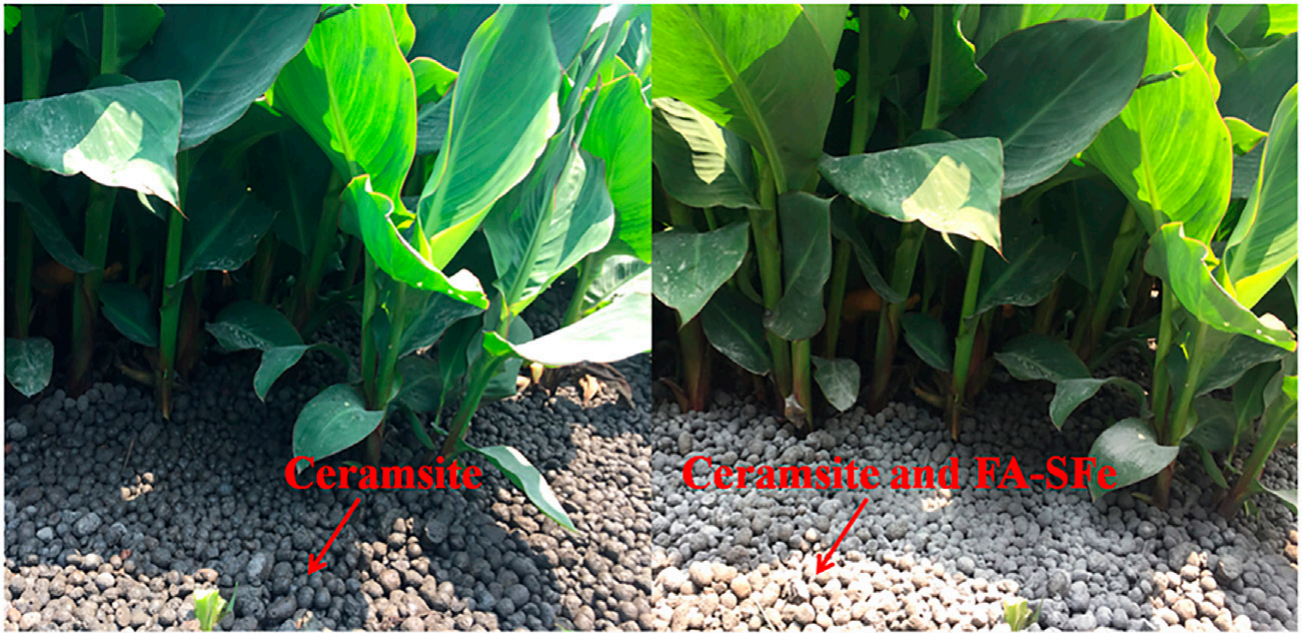
CW fillers with ceramsite and the mixture of ceramsite and FA-SFe.

Ten DSTFs containing A^2^/O + CW processes (Figure 1) were chosen for new filler application experiments. All the CWs were of similar size (6 × 8 m), plants (*Canna indica*), and raw fillers (ceramsite). The FA-SFe (500 g m^-2^) and ceramsite in CWs were mixed (volume ratio 1:5), and the new mixed fillers were used in these ten rural DSTFs (Figure 2). The influent and effluent from the ten DSTFs were collected in September 2018, and the new fillers were added to the ten CWs. After 10 months of operation, the influent and effluent were collected in June 2019.

### 2.4 Data analysis

The influent and effluent quality was determined using standard methods, and the following parameters were measured: COD (mg L^-1^), TP (mg L^-1^), P as SRP (PO_4_-P, mg L^-1^), and N as NH_3_ (NH_3_-N, mg L^-1^) (Ki et al., 2018).

The TP and NH_3_-N removal efficiency (%) of DSTFs was calculated as follows:

$$\eta =\frac{C_{i}-C_{ef}}{C_{i}}\times 100\%,$$

where *C*_*i*_ (mg L^−1^) and *C*_*ef*_ (mg L^−1^) are the pollutant concentrations of influent and effluent, respectively.

Sorption kinetics were described by pseudo-first- and -second-order models as follows (Huang et al., 2015; Huang et al., 2016):

$$Q_{t}=Q_{e}\left(1-e^{-K_{1}t}\right),$$

$$\frac{t}{Q_{t}}=\frac{1}{K_{2}Q_{e}^{2}}+\frac{t}{Q_{e}},$$

where *Q*_*t*_ and *Q*_*e*_ are the uptake amounts (mg L^−1^) of SRP or NH_3_-N adsorbed at time point *t* and equilibrium (mg g^−1^), respectively. *K*_*1*_ (h^−1^) is the first-order kinetic rate constant, and *K*_*2*_ is the sorption rate constant of the pseudo-second-order kinetic model (g mg^−1^h^−1^).

The sorption isotherms were fitted by the Langmuir and Freundlich models, and the equations of the isotherm parameters are (Sundaram et al., 2008; Chen et al., 2018):

$$Q_{e}=\frac{Q_{m}KC_{e}}{1+KC_{e}},$$

$$Q_{e}=K_{f}C_{e}^{n},$$

where *Q*_*e*_ and *Q*_*m*_ are the adsorbed amounts of SRP or NH_3_-N at equilibrium and the maximum SRP or NH_3_−N uptake amount (mg g^−1^), respectively; *C*_*e*_ is the SRP or NH_3_−N concentration in the aqueous phase at equilibrium (mg L^−1^); *K* is the affinity parameter (L mg^−1^); *K*_*f*_ is the sorption coefficient (L g ^−1^); and *n* is a constant used to measure sorption intensity or surface heterogeneity.

The pollutant surface loading (PSL, g m^-2^ d^-1^) was calculated as follows:

$$PSL=\frac{Q\left(C_{i}-C_{ef}\right)\times 10^{-3}}{A},$$

where *Q* (m^3^ d^-1^) and *A* (m^2^) are the design flow and area of CW, respectively.

### 2.5 Analytical methods

Microsoft Excel, ArcMap 10.5, and Origin 2018 software were used to analyze data and plot the graphs. The standard error (SE) was also represented with error bars from triplicate tests.

## 3 Results

### 3.1 Seasonal characterization of rural domestic sewage emission

**FIGURE 3:**
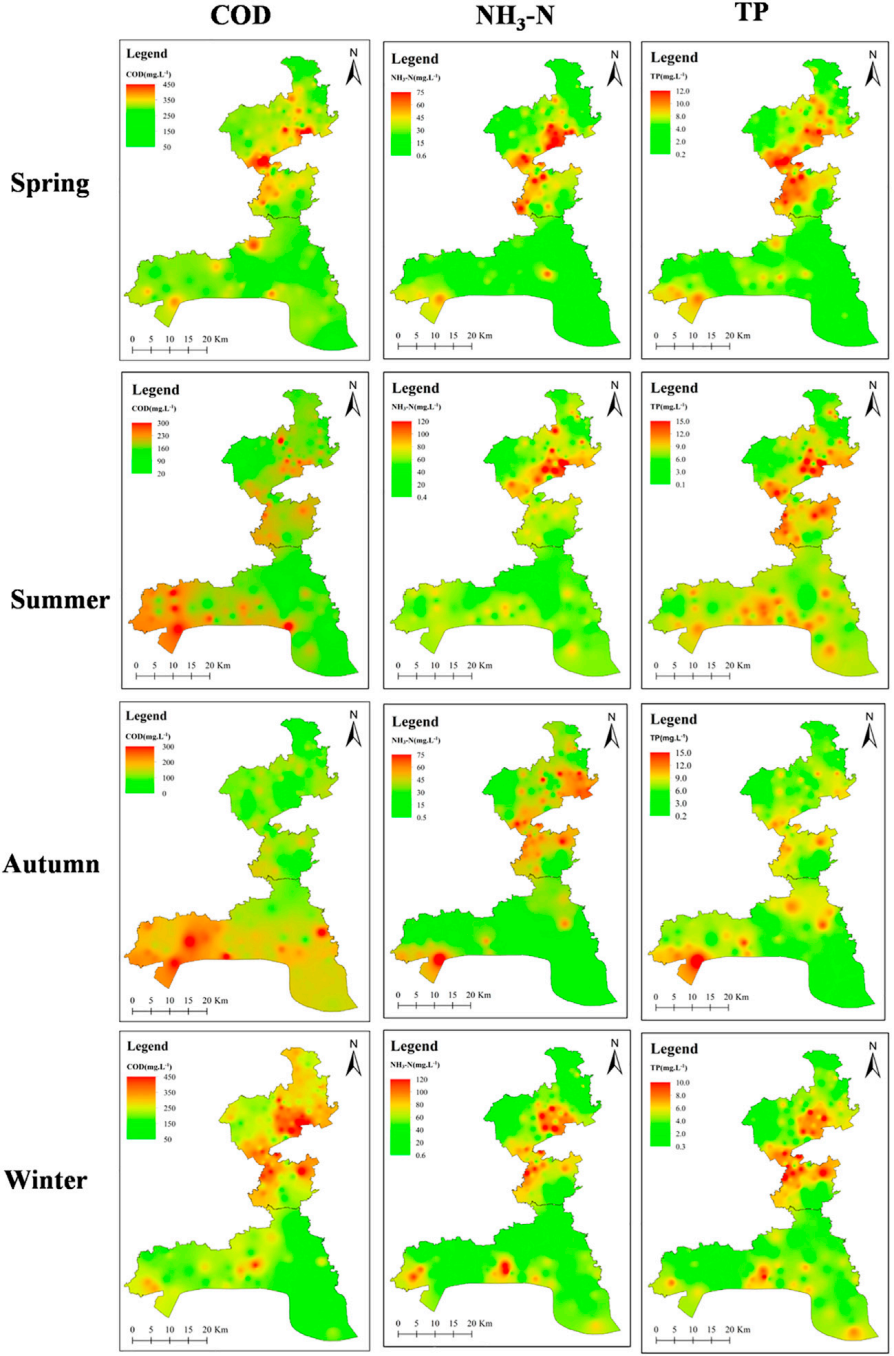
Main pollutants of influent from 160 DSTFs across four seasons.

Influent and effluent qualities of 160 DSTFs over four seasons were determined. The seasonal variation of the pollutants in influent is shown in Figure 3. The main pollutants in the influent were COD, TP, and NH_3_-N. The highest concentration of COD and NH_3_-N existed in winter, with values reaching approximately 443.8 mg L^-1^ and 73.8 mg L^-1^, respectively. The influent had the highest TP concentration (14.7 mg L^-1^) in the summer. The northern part had higher concentrations of COD, TP, and NH_3_-N than the southern part.

### 3.2 Removal efficiency of pollutants using rural DSTFs

**FIGURE 4:**
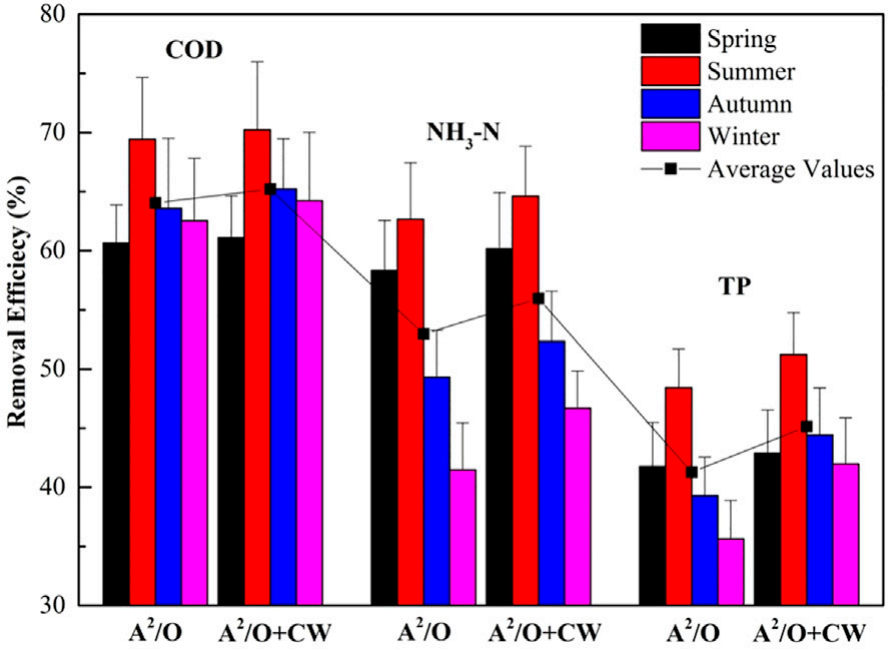
Removal efficiency of main pollutants using A^2^/O and A^2^/O + CW.

The COD, TP, and NH_3_-N removal efficiencies using A^2^/O and A^2^/O + CW in DSTFs are shown in Figure 4. A^2^/O and A^2^/O + CW had the highest COD removal efficiency, especially in summer (70.23%). TP and NH_3_-N removal efficiency presented a similar trend to COD removal efficiency, and to A^2^/O + CW in summer, which had a higher pollutant removal efficiency (64.61% of NH_3_-N and 51.23% of TP) than in other seasons. Although A^2^/O or A^2^/O + CW of DSTFs had a high pollutant removal efficiency in summer (≥50%), the pollutant removal efficiency in other seasons was relatively low, especially in winter (35.64% of TP). In addition, COD in most of the DSTFs reached the local primary standard (<60 mg/L) due to high removal efficiency using A^2^/O. However, the release of NH_3_-N and TP into RDS had difficulty reaching the local primary standard (<2 mg/L of TP, <15 mg/L of NH_3_-N). CW plays a significant role in NH_3_-N and TP removal, and the filler is one of the key factors in influencing pollutant removal in CW. Therefore, the most economical and efficient filler should be chosen and utilized in CWs.

### 3.3 Kinetics and equilibrium study of TP and NH_3_-N removal using new fillers

**FIGURE 5:**
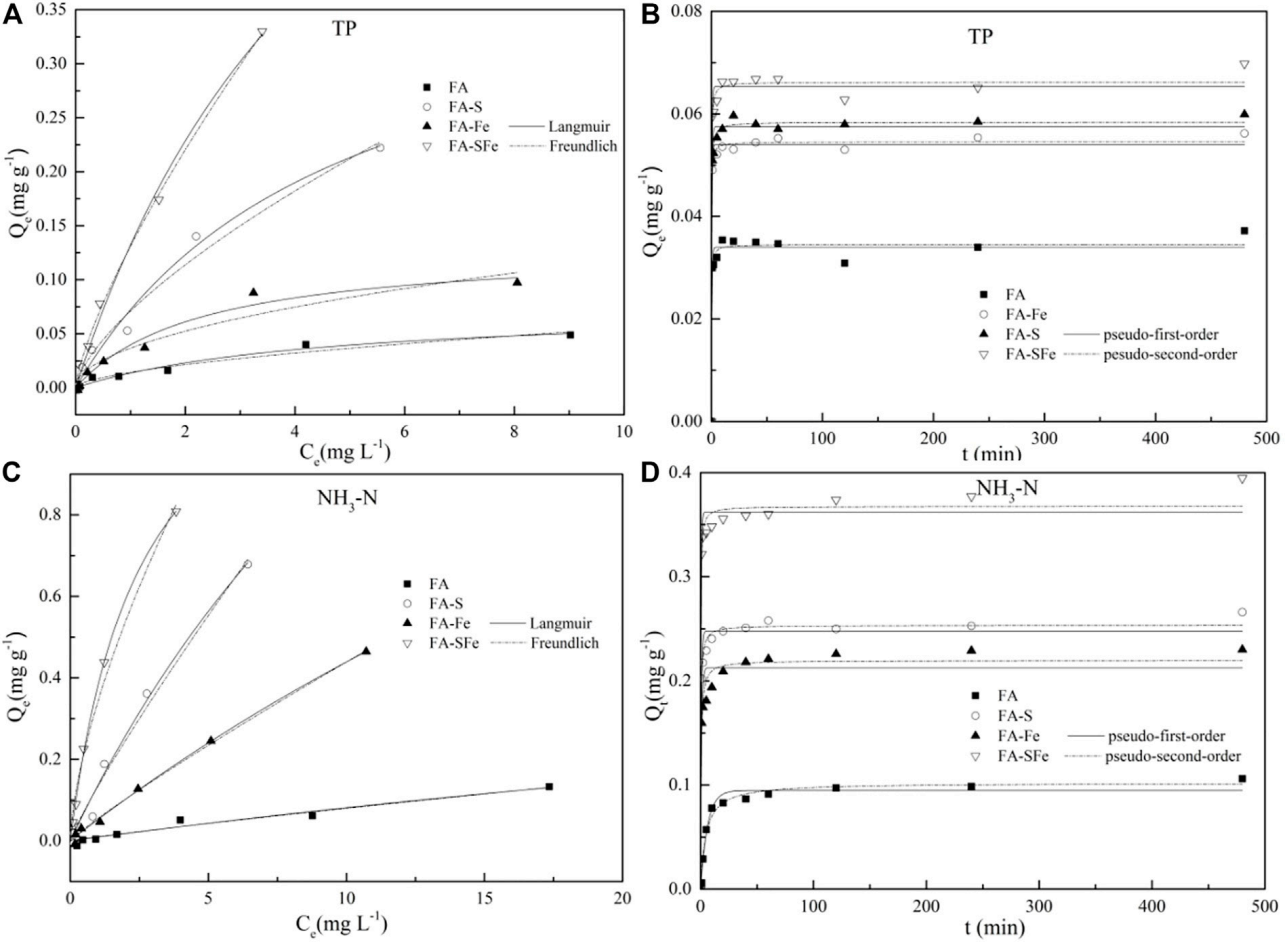
Adsorption isotherms **(A,C)** and kinetics curves **(B,D)** of phosphorus and ammonia nitrogen for four materials.

Figures 5A, C show the filler sorption isotherm, and the high correlation coefficients (*R* ^2^) indicate that the Langmuir equation is fitted better to TP and NH_3_-N with (*R* ^2^ 0.957–0.993 and 0.902–0.999, respectively) compared with the Freundlich equation (*R* ^2^ 0.902–0.996 and 0.866–0.996, respectively). The maximum sorption capacity of TP and NH_3_-N using FA-SFe could reach 0.47 mg/g and 0.91 mg/g, respectively. The kinetics of TP and NH_3_-N sorption on FA, FA-S, FS-Fe, and FA-SFe are shown in Figures 5B, C, which were fitted by the pseudo-first- and -second-order kinetic models. These results indicated that the TP and NH_3_-N sorption capacity into the fillers increased rapidly in the first 10 min of the sorption process and then increased gradually until reaching sorption equilibrium after 20 min. Comparing the different fillers, the FA-SFe had higher TP and NH_3_-N sorption capacity. In addition, the kinetic fitting results were found to better match the pseudo-second-order kinetic model than the first, and FA-SFe had a higher sorption rate of TP and NH_3_-N.

### 3.4 Application of new fillers in CWs

The new fillers (FA-SFe) were utilized in ten CWs from the DSTFs, and the indices of the CWs before and after adding new filler were determined (Table 1). In general, the performance of the CW was improved by the new fillers. The removal efficiency of NH_3_-N was increased from 64.5% to 71.3%, and PSL values were raised from 0.54 g m^3^ d^-1^–3.35 g m^3^ d^-1^. The new fillers had greatly improved TP removal, with efficiency increasing from 36.7% to 62.7%, and PSL values reaching 0.29 g m^3^ d^-1^. Compared with other CWs, these ten CWs with new fillers had the advantage of TP and NH_3_-N removal.

**TABLE 1:** TP and NH_3_-N concentrations in influent and effluent using new fillers in CWs.

Type of DSTFs	Influent (mg L^-1^)				Effluent (mg L^-1^)				Parameter			
	NH_3_-N		TP		NH_3_-N		TP		PSL (g m^-2^ d^-1^)		Efficiency (%)	
	Average	Variation	Average	Variation	Average	Variation	Average	Variation	NH_3_-N	TP	NH_3_-N	TP
Raw	22.65	0.28–119.37	1.65	0.23–5.70	8.07	0.56–72.95	1.04	0.12–4.50	0.54	0.05	64.5	36.7
New	20.37	2.37–95.80	1.58	0.19–5.65	3.33	0.4–17.55	0.59	0.08–2.17	3.35	0.29	71.3	62.7
CWs (Carstensen et al., 2019)	15.8	8.00–35	20.58	19.2–34.40	9.50	5.0–18.00	14.80	10.3–20.10	0.26	0.28	29.2	28.4
SMCWs (Li et al., 2019)	2.50	1.09–8.68	0.57	0.02–1.13	0.82	0.17–3.15	0.22	0.02–0.56	--	--	56.6	53.9
H3-CW (Bateganya et al., 2016)	1.40	--	1.10	--	0.42	--	0.54	--	2.23	1.18	70.1	50.5
H4-CW (Bateganya et al., 2016)	1.40	--	1.10	--	0.52	--	0.70	--	1.98	0.85	63.3	36.6

## 4 Discussion

### 4.1 Analysis of pollutant removal efficiency in CWs from the DSTFs

There are approximately 80,000 DSTFs in the Jiaxing rural area, of which 160 with a daily sewage treatment capacity of 30 t/d were investigated. The domestic sewage emission in the study area presented a seasonal characterization, in which COD and NH_3_-N concentrations were higher in winter than in other seasons, and TP concentration was highest in autumn. In the second half of the year, both the drop in temperature and the different living habits of residents can lead to a decrease in water consumption. This in turn leads to an increase in the concentration of COD, TP, and NH3-N in rural domestic wastewater. Temperature is a vital and contributing factor for plant growth in CWs from DSTFs in rural areas. The main performance of the influence is the change of the total phytomass and identifying the dominant plant species (Sundaram et al., 2008). NH_3_-N and TP removal relies on the sorption of the fillers in CWs and the microbial communities on plant roots and filler surfaces (Carstensen et al., 2019). In this study, *Cyperus alternifolius*, *Canna indica*, *Acorus calamus*, and *Schoenoplectus tabernaemontani* were used in the CWs, with ceramic sand, zeolite, and other natural stones utilized as fillers. The four plants adapted to the local climate, with growth conditions better in summer than in other seasons. High summer temperatures caused nitrifying and denitrifying bacterial communities to be more active, thus accelerating the adsorption rate. These transformations resulted in higher removal rates of NH3-N and TP, reaching 62.67% and 48.42%, respectively. The plants and fillers used in CWs played the principal role in TP and NH_3_-N removal in DSTFs. However, TP and NH_3_-N removal efficiency using the A^2^/O process and CWs (45.13% of TP; 55.96% of NH_3_-N) in DSTFs had no sufficient improvement compared with single A^2^/O process (41.28% of TP; 52.95% of NH_3_-N); even in summer, the removal efficiency was not greatly enhanced. The pollutant surface loading rates of 58 CWs in four seasons were calculated, and the average values of TP and NH_3_-N surface loading rates were only 0.05 and 0.54 g m^-2^ d^-1^, respectively. The TP and NH_3_-N surface loading rates generally reached 0.3–0.5 and 2–5 m^-2^ d^-1^, respectively, to ensure the high-efficiency operation of the surface flow of CWs. TP and NH_3_-N surface loading rates of the CWs from the study area were much lower than the surface flow in other CWs (Bateganya et al., 2016; Li et al., 2019), which was the main reason for the low improvement of TP and NH_3_-N removal efficiency through CWs. Therefore, the CWs in the study areas were limited by the plants and fillers used.

In addition, although the Jiaxing plain river network area is economically developed and the related treatment measures are well established, the pollutant removal efficiency of the facilities has decreased in recent years. COD removal efficiency was 64.01% on average using the A^2^/O process. The effluent quality of COD could temporarily reach the local primary standard (<60 mg L^-1^) in the study area, while NH_3_-N and TP removal efficiency of A^2^/O and A^2^/O + CW became lower. However, it was difficult for the effluent quality of NH_3_-N and TP to reach the local primary standard (<2 mg L^-1^ of TP, <15 mg L^-1^ of NH_3_-N). The lack of professional CW operation and maintenance was one of the main reasons for the low NH_3_-N and TP removal efficiency and saturated sorption or CW clogging (Smith et al., 2019). Additionally, the high proportion of SRP in TP in domestic sewage means a high demand for TP removal efficiency in CWs (Li et al., 2019). Furthermore, the Jiaxing plain river network is more economically developed compared with the central and western regions of China. The population and gross domestic product (GDP) of its rural area are larger than in other rural areas. The rural GDP in Jiaxing correlates significantly with domestic sewage emission and rural non-point source pollution, indicating that economic development influences the quality of RDS in this area, thus demanding increased operating efficiency from DSTFs.

The fillers used for CWs in this study were natural stones, which are economical and easily available, but which have low sorption ability for TP and NH_3_-N due to large particles, small specific surface area, and low porosity; this results in low removal rates for TP and NH_3_-N from CWs. Therefore, the fillers used for CWs are essential in reducing the TP and NH_3_-N concentrations in DSTF effluent.

### 4.2 Effectiveness and application of new fillers for TP and NH_3_-N

On the basis of in-depth research from 160 DSTFs in this area, it was determined that TP and NH_3_-N removal capacity could not be improved by traditional A^2^/O + CW processes. The processes investigated had low TP and NH_3_-N removal efficiency, and CWs had low PSL values. Therefore, improving the performance of fillers in CWs is key to increasing the removal efficiency of TP and NH_3_-N in RDS. Although FA has a strong P sorption capacity, it can lead to the death of plants in CWs due to its alkalinity if used as filler, as well as easily causing blockage because of its small particle size (Bateganya et al., 2016; Smith et al., 2019). The surface physical and chemical properties of FA can be changed according to acid oxidation and can be Fe-doped to improve the removal capacity of TP and NH_3_-N.

The study results indicated that the specific surface area of FA could be increased by changing the surface composition, and that FA-SFe was neutral. The surface adsorption active sites of FA-SFe rose as the modified material increased its TP and NH_3_-N removal efficiency. The adsorption equilibrium time of FA-SFe to TP and NH_3_-N is short (<30 min), which could also reduce the hydraulic retention time in its utilization in CWs. The theoretical maximum adsorption capacity of FA-SFe to TP (0.47 mg g^-1^) is higher than for ordinary natural or manufactured materials. Although the theoretical maximum adsorption capacity of FA-SFe to NH_3_-N was 0.91 mg g^-1^, FA-SFe had no great advantage compared with other adsorption materials used for NH_3_-N in CWs. The removal efficiency of TP and its content in DSTF effluent in a rural area did not reach the relevant local standards. Thus, TP became the main object of removal in the practical application of FA-SFe.

In this study, FA-SFe was added to ten DSTFs with CWs in the study area. This did not change the growth status of plants and the hydraulic load of CWs. The mixture ratio of FA-SFe and CWs was 1:5, which not only improved the adsorption capacity of CWs but also avoided blockage of CWs by the small particle size of FA-SFe. After adding FA-SFe, the PSL values of TP and NH_3_-N in CWs increased six-fold, similar to the optimal values in CWs and improving their performance. Compared with other new types like H3-CW and H4-CW in CWs, there was little difference in PSL value. In this study, after adding FA-SFe, the NH_3_-N removal efficiency of CWs slightly improved, and the TP removal rate greatly increased, mainly due to the addition of FA-SFe. Therefore, the new filler (FA-SF) had broad application prospects in CWs.

## 5 Conclusion

A new neutral filler (FA-SFe) was prepared from fly ash using acid oxidation modification and Fe ion loading. When added to CW after the conventional A^2^/O process, the maximum adsorption capacities were 0.47 mg/g and 0.91 mg/g for nitrogen and phosphorus, respectively, which could improve the deficiency of TP and NH_3_-N removal. The PSL values of TP and NH3-N were increased six-fold after adding the new filler FA-SFe to ten DSTFs with constructed wetlands in the study area—close to optimal wetlands and exceeding other novel CWs. In addition, the equilibrium time of adsorption of both TP and NH_3_-N by FA-SFe was short (<30 min) for CW filler, reducing the hydraulic residence time during actual use. Therefore, FA-SFe can be used as a new filler for CWs, significantly reducing nitrogen and phosphorus in RDS.

## Data Availability

The raw data supporting the conclusion of this article will be made available by the authors, without undue reservation.
